# Gender differences in scientific productivity: a persisting phenomenon?

**DOI:** 10.1007/s11192-012-0712-y

**Published:** 2012-04-25

**Authors:** Pleun van Arensbergen, Inge van der Weijden, Peter van den Besselaar

**Affiliations:** 1 Science System Assessment, Rathenau Institute, The Hague, The Netherlands; 2 CWTS, Leiden University, Leiden, The Netherlands; 3 Department of Organization Science & Network Institute, VU University Amsterdam, Amsterdam, The Netherlands

**Keywords:** Scholarly performance, Gender differences, Generation differences

## Abstract

There is substantial literature on research performance differences between male and female researchers, and its explanation. Using publication records of 852 social scientists, we show that performance differences indeed exist. However, our case study suggests that in the younger generation of researchers these have disappeared. If performance differences exist at all in our case, young female researchers outperform young male researchers. The trend in developed societies, that women increasingly outperform men in all levels of education, is also becoming effective in the science system.

## Introduction

**Fig. 1:**
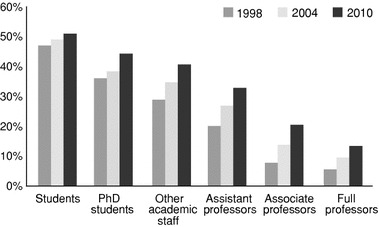
Share of women in academic positions the Netherlands 1998–2010s (source: VSNU)

The academic world has been dominated by men for a long time. However, the share of women in academia is gradually increasing. Worldwide female students nowadays even outnumber male students, with 55 % in the UK and USA and with 59 % in the Scandinavian countries (OECD 2010). And of the new entrance in European higher education about 55 % is female.^1^ Figure 1 shows the percentage of women in different academic positions in the Netherlands. There, the position of women in higher academic positions is even lower than elsewhere. The growing share of women is characteristic for all positions, however the general rule still is ‘the higher the rank in academia, the lower the number of women’ (Brouns 2000; De Weert 2001; Timmers et al. 2010). Although female researchers are improving their position, the process is rather slow. Is the weak position due to women having in average fewer ambitions in pursuing an academic career? Are career decisions characterized by gendered social closure, structurally disadvantaging women? Or are women weakly represented in high ranks because their male colleagues outperform them? In this paper we will address the last question by focusing on differences in research performance between male and female researchers.

Ample evidence has been provided for a productivity difference between men and women over time, with men producing more research output than women (Cole and Zuckerman 1984; Long 1992; Xie and Shauman 1998; Nakhaie 2002; Prpic 2002; Penas and Willett 2006; Symonds et al. 2006; Taylor et al. 2006; Ledin et al. 2007; Abramo et al. 2009). However, with regard to citations per publication no gender differences were found (Penas and Willett 2006; Ledin et al. 2007; Tower et al. 2007), or even a difference in the opposite direction; women having a higher citation score than men (Long 1992; Powell et al. 2009). The lower research productivity of women implies that female researchers receive in average a lower total number of citations than men do.

Zuckerman (2001) suggest four different types of explanations of the *productivity puzzle* (Cole and Zuckerman 1984): scientific ability, self-selection, social selection, and accumulated disadvantage. According to the scientific ability explanation, male and female academics have different biological and psychological characteristics that directly affect the research output. However, no direct gender effect has been found in earlier research (e.g. Xie and Shauman 1998).

The self-selection explanation argues that scientific productivity is influenced by the individual choices of the academics themselves. Several studies confirm the influence of individual choices. For example, women more often interrupt their career to have children and start a family (Prozesky 2008). Having children causes a decline in research productivity growth, more for women than for men (Fuchs et al. 2001; Hunter and Leahey 2010). Women were also found to initiate their careers at a later age than men (Karamessini 2004; Prozesky 2008). This also holds for their publication career: women produce fewer publications than men during the first decade of their career (Long 1992; Symonds et al. 2006), but later in their career they more or less catch up with male researchers (Long 1992; Symonds et al. 2006). Other factors which are found to affect research productivity and can be considered as self-selection are marital status,^2^ career ambitions, amount of research time, degree of specialization, discipline, reputation of the university and department, international network (collaboration and co-authoring), and academic rank (Allison and Long 1990; McNamee et al. 1990; Dundar and Lewis 1998; Prpic 2002; Lee and Bozeman 2005; Bland et al. 2006; Carayol and Matt 2006; Leahey 2006; Taylor et al. 2006; Puuska 2010). Many of these factors have a gender dimension, as women in average work at lower ranks, in less prestigious institutions, have in average less experience and a weaker (inter)national network. They also specialize less (Leahey 2006) and more often concentrate on teaching and service, and therefore spend less time on research (Taylor et al. 2006; Snell et al. 2009). However one should recognize that these factors cannot always be fully ascribed to self-selection. For example, decisions related to collaboration and academic rank are partly in the hands of other people and the organization of the university.

Zuckerman’s third type of explanation, social selection, outlines how research productivity of women is affected by gender-based decisions made by others (Zuckerman 2001). Just as in society in general, there may exist mechanisms of discrimination in the social organization of science (Prpic 2002). Men outnumber women in positions of formal power, authority and high income (Xie and Shauman 1998; Timmers et al. 2010). Research on professorial appointments shows there are gender differences in the selection and recruitment procedures. A clear disparity was found in the success rates of male and female applicants to the disadvantage of females (Van den Brink et al. 2006). This implies that career decisions are characterized by gendered social closure (Van den Brink 2009).

A similar situation has been observed in the procedures of grant allocation. Quite some research has focused on gendered aspects of peer review, especially since Wennerås and Wold (1997) published their study on nepotism and sexism in science. They showed that women needed a higher performance to be as successful as male researchers. And, researchers without committee members in their network needed much higher performance than those with an adequate network. A similar study on grant applications in the Netherlands confirmed that gender matters (Brouns 2000). However, it showed that the way it matters varies for different disciplines. Whereas in some disciplines in case of equal average publication scores more men than women were evaluated as excellent, less productive women also obtained grants in other disciplines. Replicating the study of Wenneras and Wold 10 years later, Sandstrom and Hallsten (2008) found no sexism anymore; female researchers even had a slightly better chance than males. Clearly, the council studied in both papers changed its policy in the meantime. However, nepotism was as strong as before. If that is the case, this may still influence female researchers, as male researchers generally have better networks than female researchers (Kyvik and Teigen 1996; Fuchs et al. 2001) and collaboration influences performance (Lee and Bozeman 2005). Furthermore, women receive less academic support and mentoring than men (Landino and Owen 1988; Fuchs et al. 2001). This may be a disadvantage for women too, as academic careers depend on support by academic mentors (Van Balen 2010).

The factors described above may overlap, and constitute the source of other events influencing research productivity. For example status in science can be both the cause and effect of scientific collaboration. The same holds for the relation between scientific status and publication productivity (Fox 2005). The accumulation of decisions or events over time generally placing women at a disadvantage is called cumulative disadvantage (Zuckerman 2001). However, if productivity differences relate to individual (often gendered) factors, such as ambition, focus on research, and changing gender roles and responsibilities in family life (Xie and Shauman 1998; Taylor et al. 2006; Prozesky 2008), one may expect that gradually changing gender roles in the last decades may have resulted into changed behavior.

In a recent review, (Ceci and Williams 2011) discuss the evidence about discrimination against women in science, in journal reviewing, grant funding, and in hiring. They suggest that no evidence is available that supports the current discrimination against women in science. As a consequence, the unequal position of women in science would be based on quality differences between male and female researchers that may partly be based on free choices, and partly on discriminatory arrangements in society at large—e.g., inequalities related to division of domestic work and child care. If this is correct, a careful analysis of these performance differences between male and female researchers is necessary—especially an analysis of changes in performance differences over time. We would actually expect changes, as women increasingly perform better at all levels in the educational system (Buchmann et al. 2008; Pekkarinen 2008).

## Research question

In this study, we answer the question of whether the gendered productivity differences are persistent or whether they change over time. As it was suggested that the productivity gap occurs in the early career (Symonds et al. 2006), we especially focus on the gendered performance differences among the youngest generation. Research performance in this paper is defined in terms of productivity (number of publications), and in terms of impact (number of citations).

## Materials and methods

Comparing male and female researchers requires a good identification of the population. We use data on research grant applications in the Netherlands to analyze productivity differences. The dataset^3^ covers about 1,100 applications, in a 3 years period, covering three programs: early (ECG) and advanced career grants (ACG), and an open competition scheme (OC), all within the social sciences.

1. The young career grant scheme is meant for researchers who got a Ph.D. within the previous 3 years. The grant allows them to continue to develop their ideas further.
2. The advanced career scheme is for senior researchers with a long (up to 15 years) post-doctoral experience, and who have shown the ability to successfully develop their own innovative lines of research and to act as coaches for young researchers. The grant allows them to build their own research group.
3. The open competition is for professors and senior researchers. They can apply for a 4-year full-time Ph.D. research project or a 3-year full-time postdoc project.

This set of applicants can be considered as a good representation of active social science researchers, as active researchers are expected to apply regularly in these programs. As several researchers applied two or more times during the 3 years, the number of researchers is smaller than the number of applications: 852 researchers, of which 270 (32 %) female. The advanced career applicants and the open competition applicants belong to the established generation. The young career grant is clearly for the new generation of scientists. This means we can distinguish two generations of researchers:

1. 356 young researchers, having finished their Ph.D. studies within the last 3 years;
2. 496 established researchers, generally within the associate or full professor rank.

Full and associate professors are generally older than 40, with an average of 51 years and a standard deviation of 7 years. Those with an ACG grant are on the younger side within this group: they are in average 40 years old with a standard deviation of 4 years.

The ECG grantees represent the young researchers; in our sample, they are between 27 and 41 years, with a few older: researchers who got their Ph.D. at an older age. In average, the young researchers are 33 years old, with a standard deviation of 3 years.

For this paper we define research performance as the number of articles in scholarly (peer reviewed) journals, and as the number of citations received. Research managers and science policy makers increasingly emphasize this type of output and the performance indicators based on it.^4^ More specifically, we measured scholarly performance of all researchers, in terms of publications and citations received in the *3* *years before the application*—so we take recent performance and not lifetime performance into account.

The social sciences are heterogeneous, and consist of psychology, education, pedagogy, anthropology, sociology, communication studies, geography, demography, economics and law. As publication and citation patterns differ between these fields, performance should be standardized in order to use the social and behavioral sciences as one population. However, as Table 1 shows, three fields dominate the applications: psychology, economics and law. In this paper, we therefore do the analysis first for the (unstandardized) total sample, and then repeat it for the psychology and economics individually.

**Table 1:** Applications by field and funding instrument

	ECG	OC + ACG
Psychology	87	141
Law	40	110
Economics	107	102
Sociology	27	55
Political science	12	31
Communication	6	17
Geography	12	16
Anthropology	12	9
Education	52	9
Demography	1	6
Grand total	356	496

*OC* open competition, *ACG* advanced career grant, *ECG* early career grant

## Gender differences

First of all, distribution of research performance is heavily skewed. A small number of the researchers produce the far majority of publications, and a large amount of researchers have a very small output—therefore we use non-parametric statistics.

**Fig. 2:**
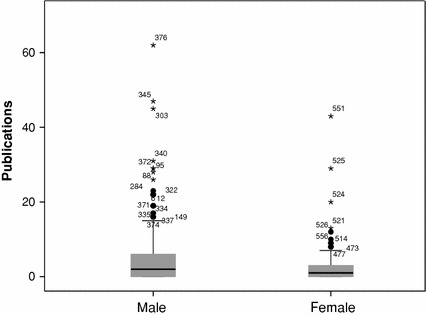
Productivity by gender, established generation social sciences, NL, 2003–2005

In the established generation, we have 496 applicants, of which about 22 % are female. In the 3 years period, male researchers did publish in average more than female researchers (mn^5^ = 4.3 publications vs. mn = 3.0). The distribution of publications by gender for the established generation (ACG and OC) is shown in Fig. 2. Clearly, the distributions are very skewed, and we test whether these distributions differ significantly. They do: (mdn^6^ = 2 vs. mdn = 1, Mann–Whitney *U* = 18666.5, *p* = 0.047).

**Fig. 3:**
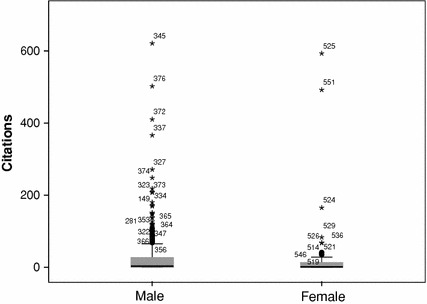
Impact by gender, established generation social sciences, NL, 2003–2005

Also in line with earlier findings, in the established generation male researchers receive more citations than female researchers do (mn = 25.9 vs. mn = 19.5). The differences are smaller than in the publications. Figure 3 presents the again skewed distributions. The difference between the distributions is significant, using a Mann–Whitney test (male: mdn = 3 vs. female: mdn = 1, *U* = 18525.5, *p* = 0.034).

## Changing gender differences?

**Fig. 4:**
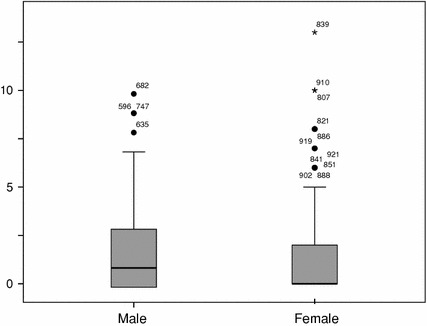
Productivity by gender, young generation social sciences, NL, 2003–2005

We repeated the analysis for the young generation (ECG applicants) with a different result. First of all, of the 356 applicants, about 45 % are female. This is a huge increase compared with the established generation (females 22 %). In the young generation of scientists, the publication differences have disappeared (Fig. 4). Male and female researchers publish in average about equal (mn = 1.7 vs. mn = 1.5). Also here we compare the distributions, but the Mann–Whitney test shows that they do not differ significantly (male: mdn = 1 versus female: mdn = 0, *U* = 14288.5, *p* = 0.126).

**Fig. 5:**
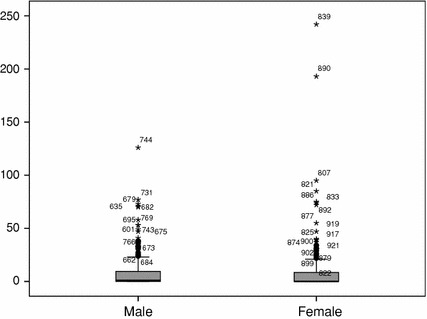
Impact by gender, young generation social sciences, NL, 2003–2005

Also the citation patterns have changed, and differences have disappeared more or less (Fig. 5). Male researchers have a higher median (mdn = 1 vs. mdn = 0) but a lower average (mn = 8.4 vs. mn = 10.5) and the Mann–Whitney test fails to show a significant difference between the distributions (*U* = 15105.5, *p* = 0.522).

Summarizing for the young researchers, in the top of the distribution (the top 7 %) women outperform men (Table 2). So if there is a gender-based difference, female researchers outperform males, especially in the top of the ranking. This result differs from what we found for the established generation and is generally found in the literature: an overrepresentation of female researchers in the lower part of the distribution, and an overrepresentation of male researchers in the higher part of the distribution. In Table 2 we also summarize the impact of the young generation by gender. In the top 10% impact ranks, female researchers are overrepresented.

**Table 2:** Performance by gender

	Established generation		Young generation	
	% Male in top	% Female in top	% Male in top	% Female in top
Top 10 % nr. publication^a^	11.6	3.6	11.2	9.4
Top ± 7 % nr. publication^b^	9.5	3.8	7.1	7.5
Top 10 % nr. citations^c^	11.2	9.4	9.2	11.3

^a^For older generation: >10 publications, for younger generation: >4

^b^For older generation: >12 publications, for younger generation: >5

^c^For older generation: >60 citations, for younger generation: >25

## A more detailed view on specific disciplines: psychology and economics

The previous analysis was done at the level of the social sciences as a whole. What if we focus on specific disciplines? We took two social science disciplines with the highest number of applications and in which English language journal articles are the main form of scholarly output.

### Psychology

Also within the group of the established psychology researchers, males (*N* = 100) in average outperform females (*N* = 41) in publications (Mn = 9.3 vs. Mn = 5.7, *p* = 0.047; Mdn = 6 vs. Mdn = 3, *U* = 1380.5, *p* = 0.002) and in citations (Mn = 73.9 vs. Mn = 46.4, *p* = 0.17; Mdn = 47 vs. Mdn = 13, *U* = 12590.0, *p* = 0.000).

The younger generation (*N* = 87) consists of more women than men, as about 55 % is female. Here, the picture is different—in line with the findings for the social sciences as a whole. Output differences have disappeared between male and female researchers (Mn = 2.31 vs. Mn = 2.48, *p* = 0.754; Mdn = 2 vs. Mdn = 2, *U* = 915.0 *p* = 0.855) in the younger generation, as have citation differences (Mn = 14.64 vs. Mn = 18.48, *p* = 0.543; Mdn = 5 vs. Mdn = 6, *U* = 880.0, *p* = 0.625).

The gender differences in the young generation are not significant, although in this case female researchers show a higher performance. As shown in Table 3, the female researchers are underrepresented in the higher part of the ranking of the established generation, but they are overrepresented in the top of the younger generation ranking.

**Table 3:** Performance by gender—psychology

	Established generation		Young generation	
	% Male in top	% Female in top	% Male in top	% Female in top
Top 10 % nr. publications^a^	11.9	5.7	7.7	12.5
Top 7.5 % nr. publications^b^	9.3	3.8	5.1	8.3
Top 10 % nr. citations^c^	11.9	5.7	7.7	10.4

^a^For older generation: >17 publications, for younger generation: >6

^b^For older generation: >20 publications, for younger generation: >7

^c^For older generation: >150 citations, for younger generation: >40

### Economics

In line with the general findings, in economics established male researchers have more publications (Mn = 3.6 vs. Mn = 1.4, *p* = 0.20; Mdn = 2 vs. Mdn = 1, *U* = 304.0, *p* = 0.169), and receive more citations (Mn = 11.5 vs. Mn = 2.1, *p* = 0.171; Mdn = 3 vs. Mdn = 0, *U* = 292.0, *p* = 0.123) than established female researchers do. The differences are considerable, however not statistically significant due to sample size.

In contrast to the psychology case, within economics young male researchers still have a higher performance than females do (publications: Mn = 1.4 vs. Mn = 0.8, *p* = 0.151; Mdn = 1 vs. Mdn = 0, *U* = 797.0, *p* = 0.012/citations: Mn = 4.7 vs. Mn = 4.2, *p* = 0.857; Mdn = 1 vs. Mdn = 0, *U* = 867.0, *p* = 0.043). But the differences have become considerably smaller as the averages show. Nevertheless, the female economists are still stronger represented than male economists in the group of low performing researchers, although less pronounced as in the older generation.

Table 4 shows that in the established generation women are not present in the in the top 10 % of the population. Yet, they are slowly entering the higher performance groups. This may suggest a similar generational trend as observed in psychology and within social sciences as a whole. If this is the case, economics clearly lags behind.

**Table 4:** Performance by gender—economics

	Established generation		Young generation	
	% Male in top	% Female in top	% Male in top	% Female in top
Top 10 % nr. publications^a^	10.7	0	11.5	3.4
Top 7.5 % nr. publications^b^	8.0	0	9.0	3.4
Top 10 % nr. citations^c^	10.7	0	11.5	3.4

^a^For older generation: >8 publications, for younger generation: >2.5

^b^For older generation: >9 publications, for younger generation: >3

^c^For older generation: >31 citations, for younger generation: >10

A factor that may explain this observation could be the relatively low share of female researchers within economics. In the established generation (*N* = 102), women are some 9 % and in the younger generation (*N* = 107) this has increased to 27 %. However, within psychology the comparable figures are 29 and 55 %.

## Conclusions and discussion

Our analysis suggests that the gendered performance differences are disappearing. In the older generation, men outperformed women in terms of publications and citations, but this is not any more the case in the younger generation. In other words, the traditional performance differences seem to disappear over time. The data even suggest that female researchers have started to outperform male researchers. This is in line with experiences in other parts of the education system, where female pupils and students are increasingly doing better than male.

This finding is significant as earlier studies found that the performance gap between male and female researchers emerged in the early career phase (Symonds et al. 2006), and exactly in this phase the differences seem to be disappearing. This also suggests that the gendered division of domestic labor, and gender differences in motivation and career planning, may be weakening. As publication and citation scores are increasingly influencing academic careers, the disappearing performance differences may be a stimulus for changing gender relations within science. Of course, the question has to be answered as whether performance differences now emerge in later phases of the research career, a question that requires additional—preferably longitudinal—research.

The current analysis is restricted to the social sciences, and it would be useful to extend the analysis to other fields, such as science, technology, engineering and medicine. Possible performance differences in these fields may be partly due to the low number of female researchers in many of these fields. However, it is also often argued that men have better math and science capacities than women, which would lead to performance differences. This question has been studied intensively, and research suggests these differences—as far as they exist—are decreasing over time (Hyde et al. 1990; EACEA 2009).

Furthermore, this study is on a west European case. As the position of women (and consequently of female researchers) differs between countries, the introduction of a cross-cultural perspective would be another useful extension.

We found differences between the different social and behavioral sciences. For psychology, we found the same patterns as for the social sciences as a whole. In economics, gendered performance differences still exists, but are much smaller in the younger generation as compared with the established generation. The performance gap is narrowing, but within economics less pronounced than within psychology. This may be related to field differences in the share of female researchers. Our study indicates that the gender distribution in the group of active social science researchers has changed considerably. In the older generation only about 22 % of the applicants are female, in the younger generation this has increased to 45 %. Within psychology, female researchers even have become the majority in the younger generation. If ‘mass’ explains performance, the remaining performance differences (in fields were the share of women is still relatively low) may disappear when women would enter those research fields in larger numbers. In those fields, efforts to increase the number of female researchers remain important.

## Footnotes

1 Of course this differs between the various fields of study. In most science, technology and engineering fields, the share of women is low.

2 Other evidence suggests that the effect of marital status is less univocal (Fox 2005).

3 The data were prepared in the context of a previous project (Van den Besselaar and Leydesdorff 2009; Bornmann et al. 2010). Coupling between publication and citation data with project data was based on name and first initial. Using information about institutional affiliation, we could distinguish persons with the same name. In a few cases of doubt, information available on their websites was used.

4 Of course, this does not cover all scientific output, let alone the societal output of researchers (De Jong et al. 2011).

5 mn  = mean.

6 mdn = median.

## References

[CR1] Abramo G, D’Angelo CA, Caprasecca A (2009). Gender differences in research productivity: A bibliometric analysis of the italian academic system. Scientometrics.

[CR2] Allison PD, Long JS (1990). Departmental effects on scientific productivity. American Sociological Review.

[CR3] Bland CJ, Center BA, Finstad DA, Risbey KR, Staples J (2006). The impact of appointment type on the productivity and commitment of full-time faculty in research and doctoral institutions. The Journal of Higher Education.

[CR4] Bornmann L, Leydesdorff L, Van den Besselaar P (2010). A meta-evaluation of scientific research proposals: Different ways of comparing rejected to awarded applications. Journal of Informetrics.

[CR5] Brouns M (2000). The gendered nature of assessment procedures in scientific research funding: The dutch case. Higher Education in Europe.

[CR6] Buchmann C, DiPrete TA, McDaniel A (2008). Gender inequalities in education. Annual Review of Sociology.

[CR7] Carayol N, Matt M (2006). Individual and collective determinants of academic scientists on productivity. Information Economics and Policy.

[CR8] Ceci SJ, Williams WM (2011). Understanding current causes of women’s underrepresentation in science. Proceedings of the National academy of Sciences of the United States of America.

[CR9] Cole JR, Zuckerman H, Maehr P, Steinkmap MW (1984). The productivity puzzle: Persistence and change in patterns of publication of men and women scientists. Advances in motivation and achievement.

[CR10] De Jong S, Van Arensbergen P, Daemen F, Van der Meulen B, Van den Besselaar P (2011). Evaluation of research in context: An approach and two cases. Research Evaluation.

[CR11] De Weert E (2001). Pressures and prospects facing the academic profession in the netherlands. Higher Education.

[CR12] Dundar H, Lewis DR (1998). Determinants of research productivity in higher education. Research in Higher Education.

[CR13] EACEA (2009). Gender differences in educational outcomes: Study on the measures taken and the current situation in Europe.

[CR14] Fox MF (2005). Gender, family characteristics, and publication productivity among scientists. Social Studies of Science.

[CR15] Fuchs S, von Stebut J, Allmendinger J (2001). Gender, science, and scientific organizations in Germany. Minerva.

[CR16] Hunter LA, Leahey E (2010). Parenting and research productivity: New evidence and methods. Social Studies of Science.

[CR17] Hyde JS, Fennema E, Lamon SJ (1990). Gender differences in mathematics performance—a metaanalysis. Psychological Bulletin.

[CR18] Karamessini M (2004). Women’s representation and progression in science careers in Greece.

[CR19] Kyvik S, Teigen M (1996). Child care, research collaboration and gender differences in scientific productivity. Science, Technology and Human Values.

[CR20] Landino RA, Owen SV (1988). Self-efficacy in university faculty. Journal of Vocational Behavior.

[CR21] Leahey E (2006). Gender differences in productivity—research specialization as a missing link. Gender and Society.

[CR22] Ledin A, Bornmann L, Gannon F, Wallon G (2007). A persistent problem—traditional gender roles hold back female scientists. EMBO Reports.

[CR23] Lee S, Bozeman B (2005). The impact of research collaboration on scientific productivity. Social Studies of Science.

[CR24] Long JS (1992). Measures of sex differences in scientific productivity. Social Forces.

[CR25] McNamee SJ, Willis CL, Rotchford AM (1990). Gender differences in patterns of publication in leading sociology journals, 1960–1985. The American Sociologist.

[CR26] Nakhaie MR (2002). Gender differences in publication among university professors in Canada. Canadian Review of Sociology and Anthropology-Revue Canadienne De Sociologie Et D Anthropologie.

[CR27] OECD. (2010). Data retrieved at October 15, 2011 from http://stats.oecd.org/Index.aspx?DataSetCode=RNENTAGE.

[CR28] Pekkarinen T (2008). Gender differences in educational attainment: Evidence on the role of tracking from a finnish quasi-experiment. Scandinavian Journal of Economics.

[CR29] Penas CS, Willett P (2006). Gender differences in publication and citation counts in librarianship and information science research. Journal of Information Science.

[CR30] Powell A, Hassan TM, Dainty ARJ, Carter C (2009). Exploring gender differences in construction research: A European perspective. Construction Management and Economics.

[CR31] Prozesky H (2008). A career-history analysis of gender differences in publication productivity among South African academics. Science Studies.

[CR32] Prpic K (2002). Gender and productivity differentials in science. Scientometrics.

[CR33] Puuska HM (2010). Effects of scholar’s gender and professional position on publishing productivity in different publication types. Analysis of a finnish university. Scientometrics.

[CR34] Sandstrom U, Hallsten M (2008). Persistent nepotism in peer-review. Scientometrics.

[CR35] Snell C, Sorensen J, Rodriguez JJ, Kuanliang A (2009). Gender differences in research productivity among criminal justice and criminology scholars. Journal of Criminal Justice.

[CR36] Symonds MRE, Gemmell NJ, Braisher TL, Gorringe KL, Elgar MA (2006). Gender differences in publication output: Towards an unbiased metric of research performance. PLoS ONE.

[CR37] Taylor SW, Fender BF, Burke KG (2006). Unraveling the academic productivity of economists: The opportunity costs of teaching and service. Southern Economic Journal.

[CR38] Timmers TM, Willemsen TM, Tijdens KG (2010). Gender diversity policies in universities: A multi-perspective framework of policy measures. Higher Education.

[CR39] Tower G, Plummer J, Ridgewell B (2007). A multidisciplinary study of gender-based research productivity in the world’s best journals. Journal of Diversity Management.

[CR40] Van Balen, B. (2010). Op het juiste moment op de juiste plaats. Waarom wetenschappelijk talent een wetenschappelijke carrière volgt. Den Haag, Rathenau Instituut.

[CR41] Van den Besselaar P, Leydesdorff L (2009). Past performance, peer review, and project selection: A case study in the social and behavioral sciences. Research Evaluation.

[CR42] Van den Brink M (2009). Behind the scenes of science: Gender in the recruitment and selection of professors in the Netherlands.

[CR43] Van den Brink M, Brouns M, Waslander S (2006). Does excellence have a gender? A national research study on recruitment and gender. Employee Relations.

[CR46] VSNU. Data retrieved at October 15, 2011 from http://www.vsnu.nl/Universiteiten/Feiten-Cijfers/Personeel/Vrouwen-in-wetenschappelijke-posities.htm.

[CR44] Wennerås C, Wold A (1997). Nepotism and sexism in peer-review. Nature.

[CR45] Xie Y, Shauman KA (1998). Sex differences in research productivity: New evidence about an old puzzle. American Sociological Review.

[CR47] Zuckerman, H. (2001). The careers of men and women scientists: Gender differences in career attainment. In: M. Wyer (Ed.) Women, science and technology: A reader in feminist science studies. Routledge: 69–78.

